# piRNA-like small RNAs are responsible for the maternal-specific knockdown in the ascidian *Ciona intestinalis* Type A

**DOI:** 10.1038/s41598-018-24319-w

**Published:** 2018-04-12

**Authors:** Teruki Satoh, Takako Iitsuka, Akira Shiraishi, Akiko Hozumi, Honoo Satake, Yasunori Sasakura

**Affiliations:** 1 Shimoda Marine Research Center, University of Tsukuba, Shimoda, Shizuoka Japan; 2 Bioorganic Research Institute, Suntory Foundation for Life Sciences, Kyoto, Japan

## Abstract

The mRNAs stored in eggs are crucial for embryogenesis. To address functions of maternal mRNAs, we recently reported the novel method MASK (maternal mRNA-specific knockdown), which we used to specifically knockdown maternal transcripts in the ascidian *Ciona intestinalis* Type A. In MASK, the *cis* element of a maternal gene is fused with *eGFP* or *Kaede* reporter gene, and the cassette is introduced into *Ciona* genome by transposon-mediated transgenesis. In eggs of the transgenic lines, the maternal expression of the gene whose *cis* element is used for driving the reporter gene is suppressed. The zygotic expression of the gene is not suppressed, suggesting that the MASK method can distinguish between maternal and zygotic functions of a gene. Here we investigated the *cis* and *trans* factors responsible for MASK results. In the ovaries in which knockdown of a maternal gene occurs, a number of antisense small RNAs are expressed that are complementary to the sequence of the knocked-down genes. We suspect that these antisense small RNAs are the factor responsible for MASK results. The antisense small RNAs have several features that are seen in PIWI-interacting RNAs (piRNAs), suggesting that MASK is likely to use a piRNA-mediated mechanism to knock down maternal mRNAs.

## Introduction

The initiative cues for animal development are given by genetic substances that are stored in eggs. The characterization of the functions of these maternal factors such as mRNAs and proteins is thus essential for understanding the mechanisms of animal development. The chordate ascidians comprise a well-known animal group, and the involvement of maternal substances on the embryogenesis of chordate ascidians was first described over a century ago^1^. Molecular studies have characterized the genes whose mRNAs function as the determinative factors for cell differentiation of ascidians^2^. Maternal factors of ascidians have various functions that are not limited to cell differentiation. These maternal factors are involved in the localization of mRNAs, unequal cleavages of blastomeres, and gastrulation^2–8^.

Many studies have revealed that a number of maternal transcripts are localized to the specific region of ascidian eggs^9–19^, suggesting that these localized RNAs are crucial for embryogenesis. However, the functions of maternally expressed genes have not been characterized well due to technical issues that are not limited to ascidians. First, the knockdown experiments in model organisms that apply antisense technologies are usually not informative for maternal proteins that are already translated during oogenesis. Second, the generation of mutant lines for maternally expressed genes requires an additional generation to observe the appearance of phenotypes compared to the investigation of zygotic functions of genes, because eggs laid by homozygous mutant females are necessary for the examination of the effect of mutations on maternal genes. Third, the generation of homozygous mutant females becomes difficult when the target maternal gene has an essential function for viability as its zygotic function, and thus the knockouts of such maternal genes by genome editing techniques or conventional knockout vectors is frequently not applicable to the phenotypes of maternal functions of the target genes without the use of a special technique such as conditional knockouts. In order to avoid these obstacles, developmental biologists have attempted to improve the methods for analyzing maternally expressed genes [e.g. ref.^20,21^].

Our group recently established a new reverse genetic method for knocking down the maternal expression of genes in the ascidian *Ciona intestinalis* Type A^22^. The method, named MASK (maternal mRNA-specific knockdown), uses an epigenetic suppression of the maternal expression of reporter genes (such as enhanced green fluorescent protein [*eGFP*] gene) from the *cis* elements of maternally expressed genes. In *Ciona*, germ-line transgenesis has been achieved with the Tc1/*mariner* superfamily transposon *Minos*^23–25^. In the transgenic lines, the *eGFP* expression in oocytes and eggs is somehow silenced by an epigenetic mechanism. When the *cis* element plus the 5′ untranslated region (5′ UTR) of a maternal gene is used to drive *eGFP*, the maternal expression of the gene is silenced together with *eGFP* in oocytes and eggs^22^. Curiously, the zygotic expression of neither *eGFP* nor the targeted maternal gene (if it has zygotic expression in *Ciona*) is silenced by MASK. This is a strong advantage for establishing mutant lines of genes that have crucial roles in both maternal and zygotic functions by a reverse genetic method, and for precisely distinguishing between maternal and zygotic functions of genes.

In light of these advantages, the use of MASK is expected to energize the studies of maternal genes in *Ciona*. A more thorough understanding of the mechanisms that underlie the MASK method will lead to the improvement of the method and to the introduction of MASK to other organisms. However, our understanding of the mechanisms of MASK is limited, and several questions remain to be solved.

In our previous report about the use of MASK^22^, we induced MASK by *eGFP*^26^ and *Kaede*^27^ reporter genes, suggesting the possibility that multiple reporter genes could be used for MASK. A deeper investigation of the reporter genes that are compatible with MASK is necessary. In a related matter, the requirement of the vector element necessary to induce MASK must be characterized in order to know how much we can modify the vector for MASK without losing the activity to knockdown maternal gene. It is also unknown how the insertion of the MASK vectors into the *Ciona* genome leads to the suppression of the maternal expression of endogenous genes. Even though the same MASK vector is used, the degree of knockdowns differs among transgenic lines, and not all of the transgenic lines harboring the MASK vector exhibit a knockdown of the target gene. The latter fact suggests that a certain manner of transgene insertion (perhaps involving the property of the inserted genomic sites or the formation of a concatemer of a transgene) may influence the occurrence of MASK. The uncertain activation of MASK is a serious disadvantage of this method, because screening for an appropriate transgenic line that shows efficient knockdown of the target gene must be conducted.

In this study, we address the characterization of *cis* and *trans* factors responsible for the knockdown of the maternal expression of genes by MASK. Our findings demonstrate that there is not a specific element in the vector that is necessary for MASK, suggesting the flexibility of vector design for this method. We also observed that small RNAs that are complementary to the target genes are expressed from the MASK vectors. Several lines of evidence suggest that the antisense small RNAs produced from the MASK vectors are the *trans* factors responsible for the knockdown of maternal expression in MASK. Based on several characteristics of the small RNAs observed in this study, we deduced the possible pathway creating the small RNAs. This information will be useful for the future improvement of MASK.

## Results

### Requirement of reporter gene for the maternal-specific knockdown

**Figure 1:**
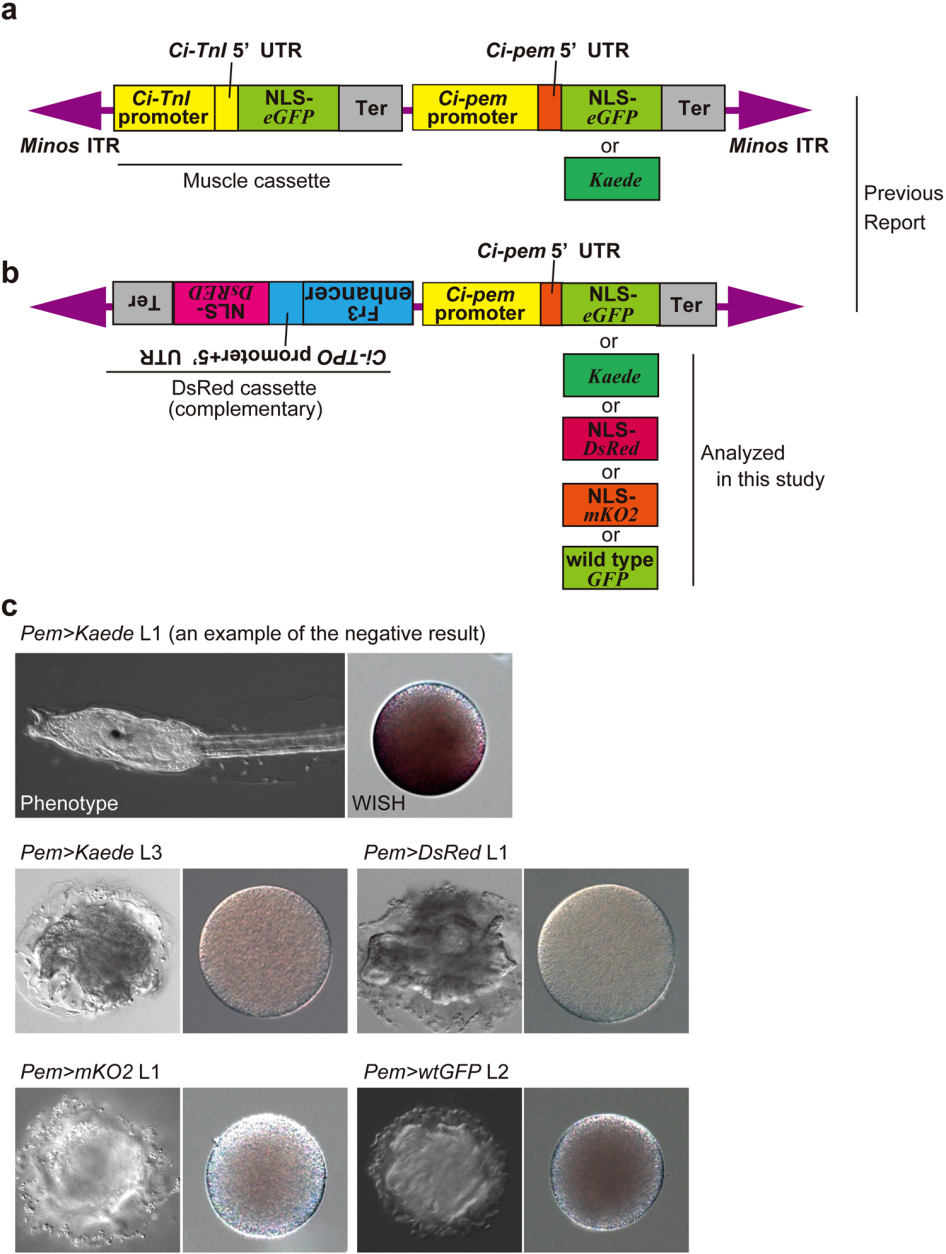
The occurrence of MASK does not rely on the primary structure of reporter gene. (**a**) The MASK vectors for knocking down *Ci-pem*. These vectors have the transcriptional cassette expressing *eGFP* in the muscle (Muscle cassette). ITR, inverted repeat of transposon; NLS, nuclear localization signal sequence; Ter, transcription termination sequence; UTR untranslated region. The colors of the genetic elements correspond to those in Fig. 3. (**b**) The MASK vector using DsRed cassette as the marker of transgenic lines. Fr3 enhancer, the enhancer isolated from the intron of *Ci-Musashi*^30^. (**c**) The knockdown of *Ci-pem*. *Left panels*: The morphology at the larval stage. Normal *Ciona* larvae exhibit the tadpole morphology. The anteroposterior axis of larvae could not be recognized in the *Ci-pem* knockdown larvae. *Right panels*: The expression of maternal *Ci-pem* mRNA in the unfertilized eggs, as revealed by *in situ* hybridization. Dark purple color corresponds to *Ci-pem* mRNA. The abbreviated names of transgenic lines indicate the reporter gene used for knocking down *Ci-pem* in the transgenic vectors shown in (**b**). *wtGFP*, *wild-type GFP*.

In a previous study, we showed that both *eGFP* and *Kaede* can induce MASK^22^. These reporter genes were driven by the *cis* elements of targeted maternal genes for inducing MASK. In addition to the MASK cassette, the 1st generation of the MASK vector contained a selectable marker cassette that drives *eGFP* in the muscle (Fig. 1a)^28^. Although we showed that the marker cassette could be changed to the one expressing DsRed, the tested vector used *eGFP* for knocking down the maternal gene (Fig. 1b). Therefore, all of the MASK vectors tested so far harbored *eGFP* as a genetic element, and this could be a shared feature inducing MASK. In the present study, we extensively analyzed whether *eGFP* is required for MASK. For this purpose, we modified the design of MASK vectors that target *Ci-pem*^10^.

First, the marker cassette that drives *DsRed* was chosen^29^ under the control of the promoter of *Ci-TPO* and the Fr3 enhancer of *Ci-Msi*^30^. Second, we exchanged the reporter gene that is driven by *Ci-pem cis* element with ones other than *eGFP*. As shown in Fig. 1b, the new vectors do not contain *eGFP*. We made transgenic lines that have the MASK vectors by using the *Minos* transposon-mediated system, and we examined whether *Ci-pem* was or was not knocked down in eggs of the transgenic animals. We observed that *Kaede*, *DsRed*, *monomeric Kusabira Orange 2* (*mKO2*)^31^, and wild-type *GFP* (the original GFP gene isolated from *Aequorea victoria*)^32^ exhibited the knockdown of *Ci-pem* (Fig. 1, Suppl. Table S1), suggesting that *eGFP* is not the genetic element required for inducing MASK. MASK can be caused by reporter genes with various DNA sequences.

### Requirement of transposon element for MASK

**Figure 2:**
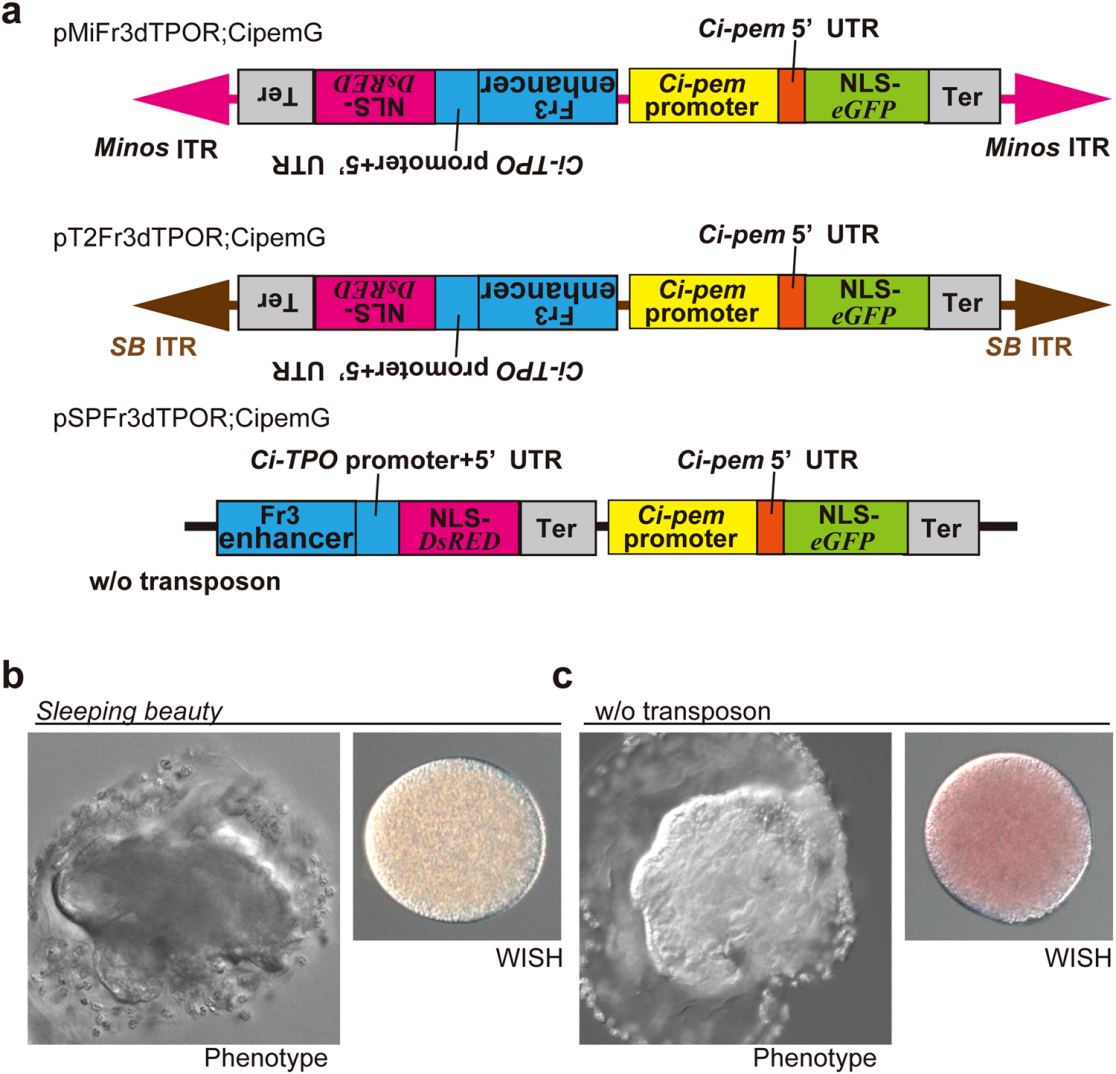
Transposon element is not required for inducing MASK. (**a**) The vectors used to analyze the necessity of transposable elements for MASK. *SB*, *sleeping beauty*. T2 indicates the improved isoform of *SB*. (**b**) Occurrence of *Ci-pem* knockdown in eggs of the transgenic line created by the *sleeping beauty* transposon vector. (**c**) Occurrence of *Ci-pem* knockdown in eggs of the transgenic line created by the vector (pSPFr3dTPOR;CipemG). Which does not have a transposable element.

Because the DNA sequence of the reporter gene is not confined to a specific gene for inducing MASK, we next attempted to characterize the vector element other than reporter gene that is necessary for MASK. Because transposable elements are the representative target of epigenetic silencing^33^, we examined whether *Minos* transposon element is necessary for MASK. In our previous research, we introduced *Sleeping Beauty* (*SB*) transposon-based transgenesis in *Ciona*^34–36^. We used *SB* instead of *Minos* to create transgenic lines for the knockdown of *Ci-pem* by MASK (Fig. 2a). Two transgenic lines were created, and one exhibited the knockdown of *Ci-pem* (Fig. 2b), suggesting that *Minos* is not the factor responsible for MASK.

To further examine the necessity of transposon element for MASK, we created transgenic lines without transposon elements. We showed previously that the germ-line transformation of *Ciona* can be achieved by the electroporation of plasmid vectors that do not include a transposon element^37^. By this electroporation-mediated method, we created two transgenic lines of the vector, pSPFr3dTPOR;CipemG, which did not have a transposon element (Fig. 2a). Among them, one transgenic line exhibited the knockdown of *Ci-pem* in eggs (Fig. 2c), suggesting that transposon element is not necessary for inducing MASK. Overall, our results showed that transposable element is not required for MASK, confirming that the vector design can be changed flexibly in this method.

### Small RNAs complementary to target mRNA are expressed in the ovary of MASK lines

**Figure 3:**
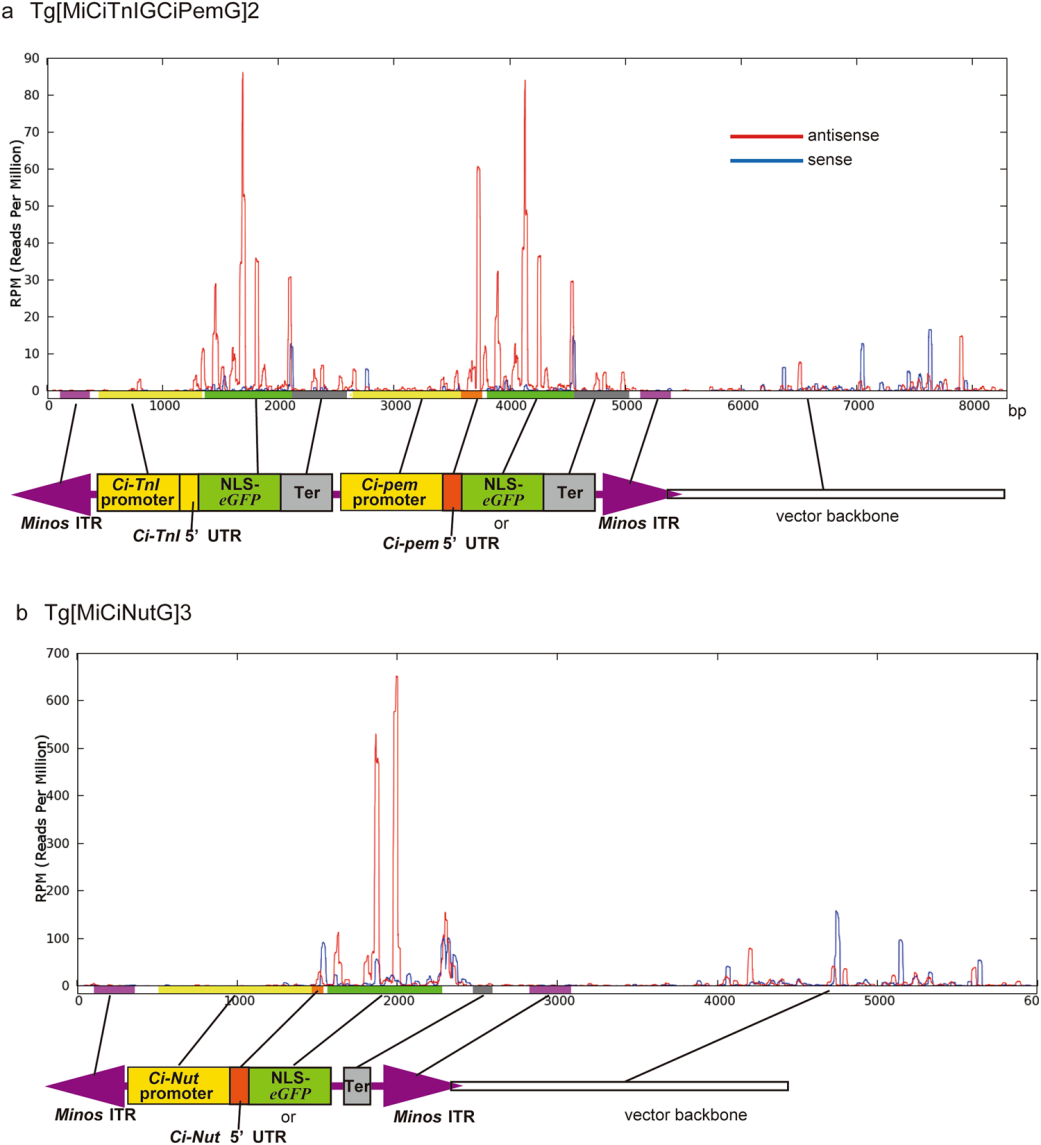
Small RNAs that have the homologous sequence to MASK vectors are formed in the ovary of MASK transgenic lines. (**a**) The small RNAs found in the ovary of Tg[MiCiTnIGCipemG]2. The number at the bottom is the length of the vector in base pairs (bp). The graph shows the number of times (read per million) each nucleotide in the vector appears in the sequenced small RNAs. The red and blue bars in the panels correspond to the results of antisense and sense strands, respectively. The colors correspond to the genetic elements in the vector. The MASK vector contains two identical copies of NLS-*eGFP*, Ter, and *Minos* ITRs elements, and we could not distinguish which copy the small RNAs corresponding to the elements are derived from. Therefore, these elements have the same peak patterns between the two copies. (**b**) The small RNAs found in the ovary of Tg[MiCiNutG]3.

In MASK, the location of the MASK vector in the genome is usually not close to the genomic locus where the target gene is encoded^22^. A factor created from the MASK vector is thus suspected to reach the target gene or its mRNA to suppress it. To examine whether such a *trans* factor is present, we sequenced small RNAs isolated from ovaries of the MASK lines, because small RNAs are good candidates for the epigenetic silencing of genes^38–40^. Small RNAs isolated from the ovary of the MASK transgenic line Tg[MiCiTnIGCipemG]2 (this line exhibits silencing of *eGFP* and *Ci-pem* in all eggs^22^) were sequenced, and the small RNAs homologous to the DNA sequence of the *Ci-pem* MASK vector were mapped onto the vector (Fig. 3a).

The results revealed the extensive expression of small RNAs that are complementary to the protein coding region (the ORF) of *eGFP*. In addition, small RNAs that are complementary to the 5′ UTR of *Ci-pem*, the required element for the knockdown of *Ci-pem*^22^, were also extensively expressed (Fig. 3a, Suppl. Table S2). Small RNAs that correspond to the sense strands of *eGFP* ORF or *Ci-pem* 5′ UTR were also identified; however, their quantities are much lower than those of antisense small RNAs (blue vs. red lines in Fig. 3a). The peaks of the small RNAs are accumulated around the genetic elements of the vector which is transcribed from the *cis* elements (Fig. 3), suggesting the necessity of transcription to the production of small RNAs.

The quantities of antisense small RNAs corresponding to the target genes of MASK correlate with the degree of the knockdown of target genes in different MASK lines. A lesser production of *eGFP* and *Ci-pem* 5′ UTR antisense small RNAs was observed in the ovary of Tg[MiCiTnIGCipemG]1 compared to that of Tg[MiCiTnIGCipemG]2 (Suppl. Table S2). Tg[MiCiTnIGCipemG]1 exhibited the knockdown of *eGFP* and *Ci-pem* in an imperfect manner (some of the eggs of this line escaped from the knockdown of these genes)^22^. Moreover, when we sequenced small RNAs expressed in the ovary of the transgenic line Tg[MiCiTnIGCipemG]9, which has the same transgene as Tg[MiCiTnIGCipemG]2 but does not exhibit the knockdown of *eGFP* or *Ci-pem*^22^, antisense small RNAs corresponding to *eGFP* and the *Ci-pem* 5′ UTR were barely found (Suppl. Table S2).

We sequenced the small RNAs isolated from the somatic tissue (mantle layer) of Tg[MiCiTnIGCipemG]2, where MASK does not occur^22^. This experiment showed that the expression of the small RNAs corresponding to *Ci-pem* 5′ UTR was not detected in the somatic tissue of the MASK transgenic line, whereas a considerable amount of small RNAs corresponding to the ORF of *eGFP* was expressed in the mantle layer (Suppl. Table S2, Tg[MiCiTnIGCipemG]2 mantle). The small RNAs for the *eGFP* ORF expressed in the mantle of Tg[MiCiTnIGCipemG]2 were very short (they have a peak around 6–10 nt long; Table 1), and they may not have the activity to suppress *eGFP*, as we discuss later regarding the length of small RNAs effective for the knockdown in MASK.

**Table 1:** The length of small RNAs.

Length in nucleotides	% of small RNAs																						
	Tg[MiCiTnIGCipemG]2 ovary					Tg[MiCiTnIGCipemG]1 ovary					Tg[MiCiTnI-GCipemG]2 mantle		Tg[MiFr3dTPORCipemK]4 ovary					Tg[MiCiNutG]3 ovary					
	*eGFP* ORF		*Cipem* 5′UTR		*Cipem* ORF	*eGFP* ORF		*Cipem* 5′UTR		*Cipem* ORF	*eGFP* ORF		*Kaede* ORF		*Cipem* 5′UTR		*Cipem* ORF	*eGFP* ORF		*Nut* 5′UTR		*CNut* ORF	
	sense	**anti-sense**	sense	**anti-sense**	anti-sense	sense	**anti-sense**	sense	**anti-sense**	anti-sense	sense	anti-sense	sense	**anti-sense**	sense	**anti-sense**	anti-sense	sense	**anti-sense**	sense	**anti-sense**	sense	anti-sense
1–5	0	**0**	0	**0**	0.5	0	**0**	0	**0**	0	0	0	0	**0**	0	**0**	0	0	**0**	0	**0**	0	0
6–10	0	**0**	0	**0.2**	2	0	**0**	0	**0**	0	56.5	92.6	0.6	**0**	0	**0**	0	0.5	**0**	0	**0**	0.6	5.8
11–15	3.1	**0.6**	0	**0**	0.2	0	**0**	0	**0**	0	34.7	2	0	**1.2**	0	**0**	0	2.5	**1**	0	**0**	0	2.5
16–20	13.6	**0.4**	0	**0.8**	1	25	**1**	0	**0**	16.6	0	0	2	**1.2**	0	**0**	0	16.4	**1.3**	0.9	**5.4**	1	13.3
21–25	42.1	**7.2**	100	**2.5**	2.5	25	**0**	0	**0**	0	4.3	0	32.6	**15.1**	0	**28.5**	33.3	21.8	**22.4**	13.3	**33.6**	18.9	25.8
26–30	36.8	**84.8**	0	**91.7**	89.1	50	**91.3**	0	**100**	83.3	0	0.6	60.6	**74.6**	0	**71.4**	66.6	46.3	**66.2**	54.9	**50**	55.8	44.1
31–35	4.2	**6.7**	0	**4.5**	4.5	0	**7.6**	0	**0**	0	0	0	4	**6.9**	0	**0**	0	5.5	**7.7**	28.8	**3.2**	21.4	2.5
36-	0	**0**	0	**0**	0	0	**0**	0	**0**	0	4.3	4.6	0	**0.6**	0	**0**	0	6.7	**1**	1.9	**7.6**	2.1	5.8
Average length	23.8	**28.2**	24	**28.4**	27.8	24.5	**28.5**	0	**28.1**	26.5	11.9	9.8	25.9	**27.3**	0	**26**	26.3	26.3	**27.4**	28.8	**26.5**	28	24.2
No. of read small RNAs	95	**1075**	1	**350**	395	4	**92**	0	**7**	6	46	149	150	**158**	0	**7**	3	948	**4391**	315	**92**	476	120

*The scores corresponding to the antisense small RNAs that are thought to be responsible for MASK are shown in bold.

To further investigate whether the antisense small RNAs are the substance responsible for MASK, we sequenced small RNAs isolated from the transgenic line (Tg[MiCiNutG]3), which exhibits maternal knockdowns of both *eGFP* and *Ci-Nut*^41^ but not *Ci-pem*^22^. The ovary of Tg[MiCiNutG]3 expressed abundant antisense small RNAs for *eGFP* and the *Ci-Nut* 5′ UTR (Fig. 3b, Suppl. Table S2). This transgenic line also exhibited abundant expression of sense small RNAs for the 5′ UTR of *Ci-Nut*. Expression of the antisense small RNAs for *eGFP* and the *Ci-Nut* 5′ UTR was not seen in the ovary of the other transgenic line Tg[MiCiNutG]4, which has the same transgene as Tg[MiCiNutG]3 but does not show the knockdowns of *eGFP* or *Ci-Nut* (Suppl. Table S2)^22^. In both Tg[MiCiNutG]3 and Tg[MiCiNutG]4, the antisense small RNAs for the *Ci-pem* 5′ UTR were not seen, suggesting that the creation of antisense small RNAs for a target gene is dependent on the transgene. In conclusion, the production of antisense small RNAs coincides with the occurrence and degree of the knockdown of target genes, suggesting that the small RNAs are the substance responsible for MASK.

### The characteristics of MaskRNAs

**Figure 4:**
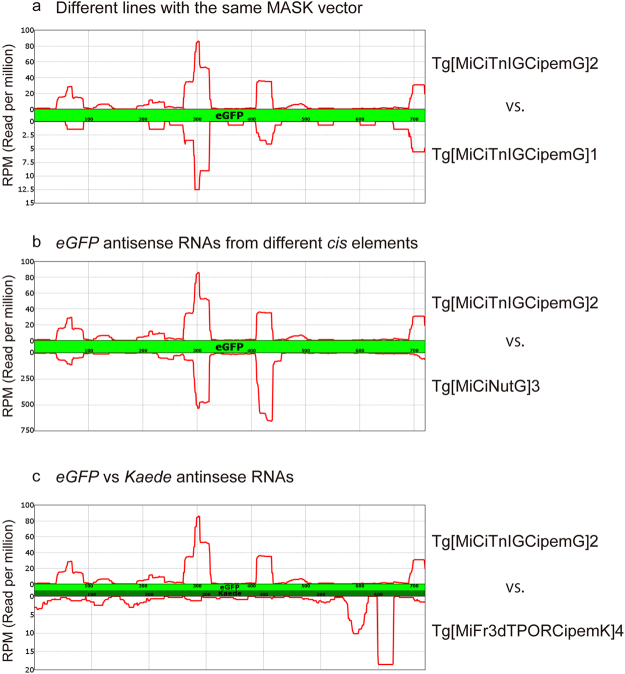
The comparisons of antisense small RNAs corresponding to the reporter genes. To enhance the visibility, the scales of the vertical bar (corresponding to RPM) are not the same between the graphs. (**a**) Comparison of *eGFP* antisense small RNAs between Tg[MiCiTnIGCipemG]2 and Tg[MiCiTnIGCipemG]1. (**b**) Comparison of *eGFP* antisense small RNAs between Tg[MiCiTnIGCipemG]2 and Tg[MiCiNutG]3. (**c**) Comparison of *eGFP* and *Kaede* antisense small RNAs between Tg[MiCiTnIGCipemG]2 and Tg[MiFr3dTPORCipemK]4. Because *eGFP* and *Kaede* have similar but different nucleotide lengths, the horizontal axis is somewhat different between the two results.

The region of the *eGFP* ORF from which *eGFP* antisense small RNAs are likely to originate is similar between the antisense small RNAs in the ovaries of Tg[MiCiTnIGCipemG]2 and Tg[MiCiTnIGCipemG]1 (Fig. 4a). Moreover, the *eGFP* antisense small RNAs in the ovaries of Tg[MiCiTnIGCipemG]2 and Tg[MiCiNutG]3 are also formed from similar *eGFP* regions (Fig. 4b), even though these two transgenic lines drive *eGFP* from different *cis* elements. These data suggest that antisense small RNAs for *eGFP* are formed through a related process in the MASK transgenic lines that is dependent on the primary structure of *eGFP*.

To further examine whether the position of antisense small RNAs is dependent on the sequence of the gene and is independent of the maternal *cis* element that drives reporter genes, we sequenced the small RNAs isolated from the ovary of Tg[MiFr3dTPORCipemK]4 (Suppl. Table S3). This transgenic line uses *Kaede* reporter gene for knocking down *Ci-pem*. Unlike *eGFP* antisense small RNAs (which are located mostly in the 5′ half of the *eGFP* ORF), the antisense small RNAs for *Kaede* were preferentially derived from the region near the 3′ end of *Kaede* ORF (Fig. 4c). Therefore, the location of the antisense small RNAs is not determined by the *cis* element adjacent to the reporter gene. Rather, the location of the antisense small RNAs created in the MASK lines is likely to be dependent on the transcribed sequences.

The length of the antisense small RNAs corresponding to the *eGFP* and *Ci-pem* 5′ UTR has a peak that is approx. 26–30 nucleotide (nt) long, and their average lengths are 26.0–28.5 nt (Table 1, Suppl. Table S4). The length of the antisense small RNAs is similar to that seen in piRNAs^42,43^. Moreover, the 5′ end of these antisense RNAs is likely to be uridine (U; Table 2). This characteristic is also seen in piRNAs. The antisense small RNAs are likely to be responsible for MASK, and we thus named them MaskRNAs. The definition of MaskRNAs is: (1) derived from the antisense strand of targeted genes of MASK, (2) the length is approx. 26–30 nt long, and (3) the 1st nucleoside is preferentially U.

**Table 2:** 1st nucleotides of small RNAs.

	% of small RNAs																						
1st base	Tg[MiCiTnIGCipemG]2 ovary					Tg[MiCiTnIGCipemG]1 ovary					Tg[MiCiTnI-GCipemG]2 mantle		Tg[MiFr3dTPORCipemK]4 ovary					Tg[MiCiNutG]3					
	*eGFP* ORF		*Cipem* 5′UTR		*Cipem* ORF	*eGFP* ORF		*Cipem* 5′UTR		*Cipem* ORF	*eGFP* ORF		*Kaede* ORF		*Cipem* 5′UTR		*Cipem* ORF	*eGFP* ORF		*Nut* 5′UTR		*Nut* ORF	
	sense	**anti-sense**	sense	**anti-sense**	anti-sense	sense	**anti-sense**	sense	**anti-sense**	anti-sense	sense	anti-sense	sense	**anti-sense**	sense	**anti-sense**	anti-sense	sense	**anti-sense**	sense	**anti-sense**	sense	anti-sense
A	21	**4.4**	100	**2.8**	3.2	25	**0**	0	**0**	0	19.5	0.6	42	**8.8**	0	**14.2**	0	17	**2.1**	18.7	**9.7**	13.6	10
U	45.2	**87.4**	0	**96.2**	93.4	25	**94.5**	0	**71.4**	83.3	6.5	18.1	46.6	**75.9**	0	**85.7**	100	48.9	**89.6**	64.1	**79.3**	71.6	79.1
C	8.4	**3**	0	**0.2**	2.2	0	**0**	0	**0**	0	23.9	9.3	6	**3.1**	0	**0**	0	10.5	**2.6**	0.3	**5.4**	1	4.1
G	25.2	**5**	0	**0.5**	1	50	**5.4**	0	**28.5**	16.6	50	71.8	5.3	**12**	0	**0**	0	23.4	**5.5**	16.8	**5.4**	13.6	6.6
No. of small RNAs	95	**1075**	1	**350**	395	4	**92**	0	**7**	6	46	149	150	**158**	0	**7**	3	948	**4391**	315	**92**	476	120

*The scores in bold correspond to the antisense small RNAs that are thought to be responsible for MASK.

We identified the expression of antisense small RNAs that can target the ORF of *Ci-pem* in the ovary of Tg[MiCiTnIGCipemG]2, Tg[MiCiTnIGCipemG]1 and Tg[MiFr3dTPORCipemK]4 (Suppl. Tables S2, S3). Likewise, antisense small RNAs that can target the ORF of *Ci-Nut* were also observed in the ovary of Tg[MiCiNutG]3; in the case of Tg[MiCiNutG]3, sense small RNAs that correspond to the ORF of *Ci-Nut* are more abundant than those for the antisense strand. Because ORFs of *Ci-pem* or *Ci-Nut* are not included in the MASK vectors, the presence of the antisense small RNAs of these ORFs suggests that the antisense strands of these ORFs are somehow transcribed in the transgenic lines. Like MaskRNAs, the antisense small RNAs corresponding to the ORFs of targeted genes tend to start with U, and tend to be approx. 26–30 nt long (Tables 1 and 2), suggesting that these antisense small RNAs for ORFs are formed via a process that is similar to that of MaskRNAs.

### MASK induces the degradation of target mRNA

There are several processes of target gene downregulations by antisense small RNAs including piRNAs^42^; i.e., transcriptional silencing, the blocking of translation without degradation, and the degradation of target RNAs. To investigate which mechanism is used in MASK, we observed *eGFP* mRNA microinjected into eggs derived from MASK-positive and MASK-negative (control) animals. To mimic the occurrence of MASK, *eGFP* mRNA was microinjected into unfertilized eggs and then the eggs were subjected to fertilization to develop until the tailbud stage. We observed the fluorescence of eGFP expressed in the tailbud embryos, and detected *eGFP* mRNAs in the embryos by means of *in situ* hybridization.

**Figure 5:**
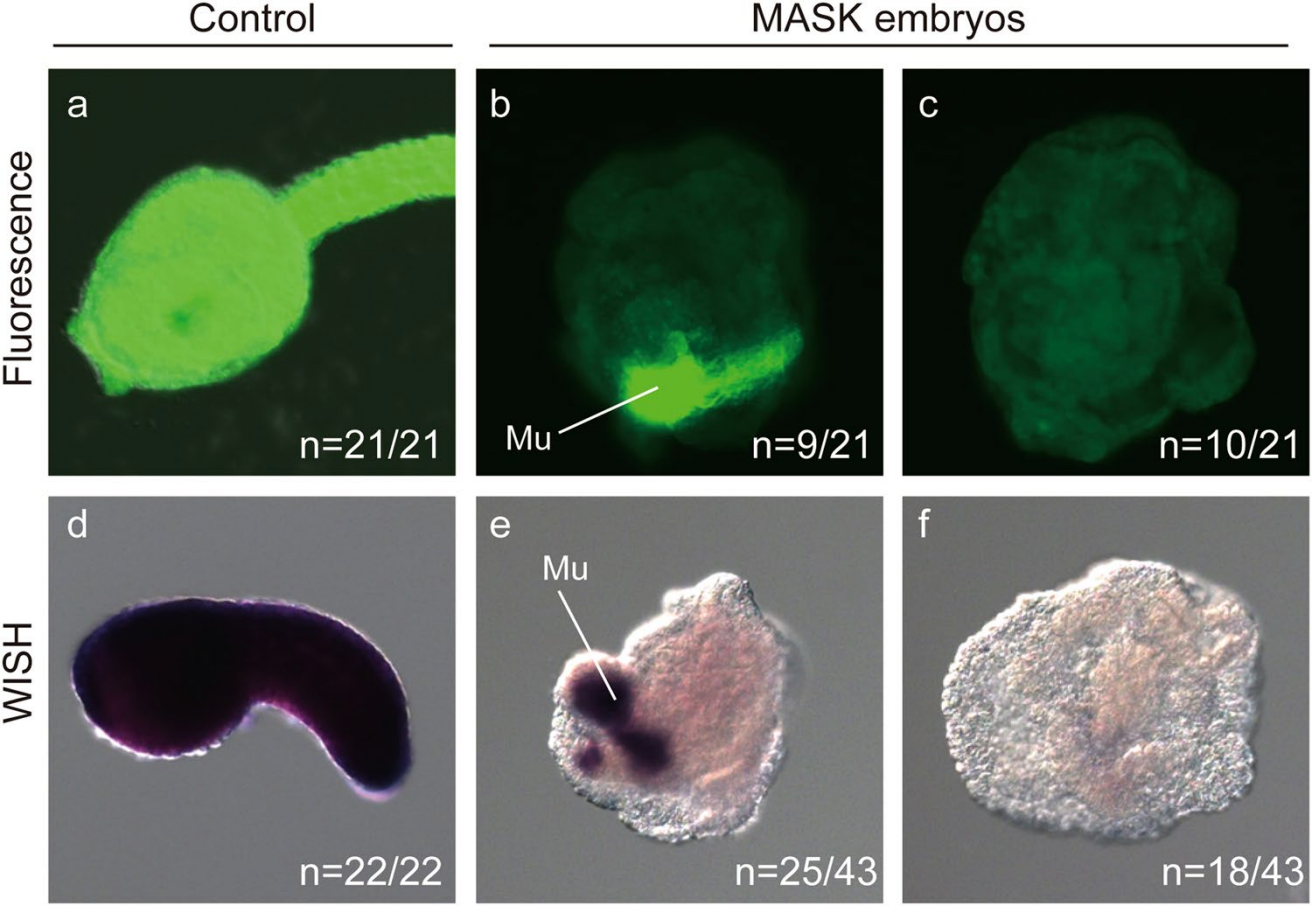
MASK induces the degradation of mRNA. All displayed embryos are derived from eggs in which *eGFP* mRNA was microinjected before fertilization. (**a**–**c**) Pseudocolored fluorescent images of eGFP. (**d**–**f**) *eGFP* mRNA detected by means of *in situ* hybridization. Dark purple color illustrates the presence of *eGFP* mRNA. (**a**–**c**) Late tailbud embryos. (**d–f**) Early tailbud embryos. (**a,d)** Embryos developed from the cross of wild-type eggs × sperm of Tg[MiCiTnIGCipemG]2. (**b,c,e,f**) Embryos developed from the cross of wild-type sperm × eggs of Tg[MiCiTnIGCipemG]2. Mu, *eGFP* signal derived from the zygotic expression in the muscle lineage.

The results revealed that the injected *eGFP* mRNA remained to be translated throughout the body of the control animals developed from wild-type eggs × sperm of the MASK transgenic line (Tg[MiCiTnIGCipemG]2), whereas the mRNA was almost abolished in embryos developed from wild-type sperm × eggs of the MASK transgenic line (Fig. 5). This suggests that *eGFP* mRNA is degraded in MASK eggs. Because we keep the MASK transgenic line as hemizygous animals, about one-half of the examined embryos possessed *TnI* > *eGFP* cassette that expresses *eGFP* zygotically in the muscle cells. The zygotic *eGFP* in the muscle was not suppressed in embryos developed from MASK eggs (Fig. 5b,e). In order to examine whether muscle cells have the ability to suppress maternal *eGFP* mRNA, we observed the *eGFP* expression of *TnI* > *eGFP* negative embryos into which *eGFP* mRNA was microinjected before fertilization. The embryos exhibited a reduction of injected *eGFP* mRNA in all blastomeres including muscle (Fig. 5c,f), suggesting that the muscle cell lineage possesses the ability to downregulate *eGFP* supplied before fertilization. Therefore, *eGFP* mRNA expressed in a zygotic manner can somehow escape from MASK.

## Discussion

In the present study, we addressed how MASK, the maternal specific knockdown, occurs in *Ciona* to obtain clues for the future improvement of this technique. Our findings demonstrated that there is not a specific DNA stretch in the MASK vectors that is necessary to induce knockdown. Small RNAs that have sequences that are complementary to the target genes are abundantly produced in the ovaries of MASK transgenic lines. Considering that the production of small RNAs is concentrated on the DNA stretches transcribed from the maternal *cis* element of MASK vectors, the requiring characteristic of MASK vectors is the *cis* element that can drive reporter gene expression in the maternal fashion. It is likely that the sense and antisense RNAs transcribed from the maternal *cis* element are used as the seeds to create MaskRNAs (see the discussion below). This transcription-dependent hypothesis can explain the lack of the need for a specific DNA sequence in the MASK vector for inducing MASK.

We observed that antisense small RNAs that are suspected to downregulate target gene are produced in the ovaries of MASK transgenic lines. Among them, we defined MaskRNAs as the small RNAs that have characteristics similar to those of PIWI-interacting (pi)RNAs^42–44^. Indeed, the length of the major antisense small RNAs produced in the ovaries of MASK lines is approx. 26–30 nt long. Typical microRNAs that are known to act to regulate gene expression have uniform lengths, i.e., approx. 21–22 nt^45^, suggesting that the mechanism underlying the production of MaskRNAs is likely to be different from the mechanism for producing microRNAs. The length of 26–30 nt coincides with the characteristic of piRNAs. The 5′ terminals of antisense piRNAs are preferentially U. The major antisense small RNAs produced in the ovaries of MASK lines also preferentially have U at their 5′ end.

If we assume that MaskRNAs are a type of piRNAs, several characteristics of MASK can be explained. piRNAs are the major RNAs that function in the post-transcriptional silencing of transposons in the gonad, and based on this characteristic, piRNAs are usually expressed specifically in the gonad in mouse^46–48^. The specificity of piRNAs in the gonad coincides with the finding that MASK occurs specifically in oocytes and eggs; the zygotic expression of target genes is not suppressed by MASK. piRNAs are amplified by a ‘ping-pong’ mechanism that requires both sense and antisense strands of the transcripts of target genes (and creates both sense and antisense small RNAs)^49^. As we stated above in the Results section, the formation of MaskRNAs is concentrated on the maternal transcriptional unit in the examined MASK vectors. This can be explained if we assume that MaskRNAs are produced via the transcription-dependent ping-pong mechanism. We suspect that the requirement of a part of the target gene (in the case of our previous study, the 5′ UTR^22^) in the transcription unit exists because the genetic element serves as the seed to create piRNAs that can target the target maternal mRNAs. piRNAs are known to cause the degradation of target transcripts^43^. Our present findings demonstrated that mRNA degradation is a major mechanism of the downregulation of maternal transcripts in MASK.

**Figure 6:**
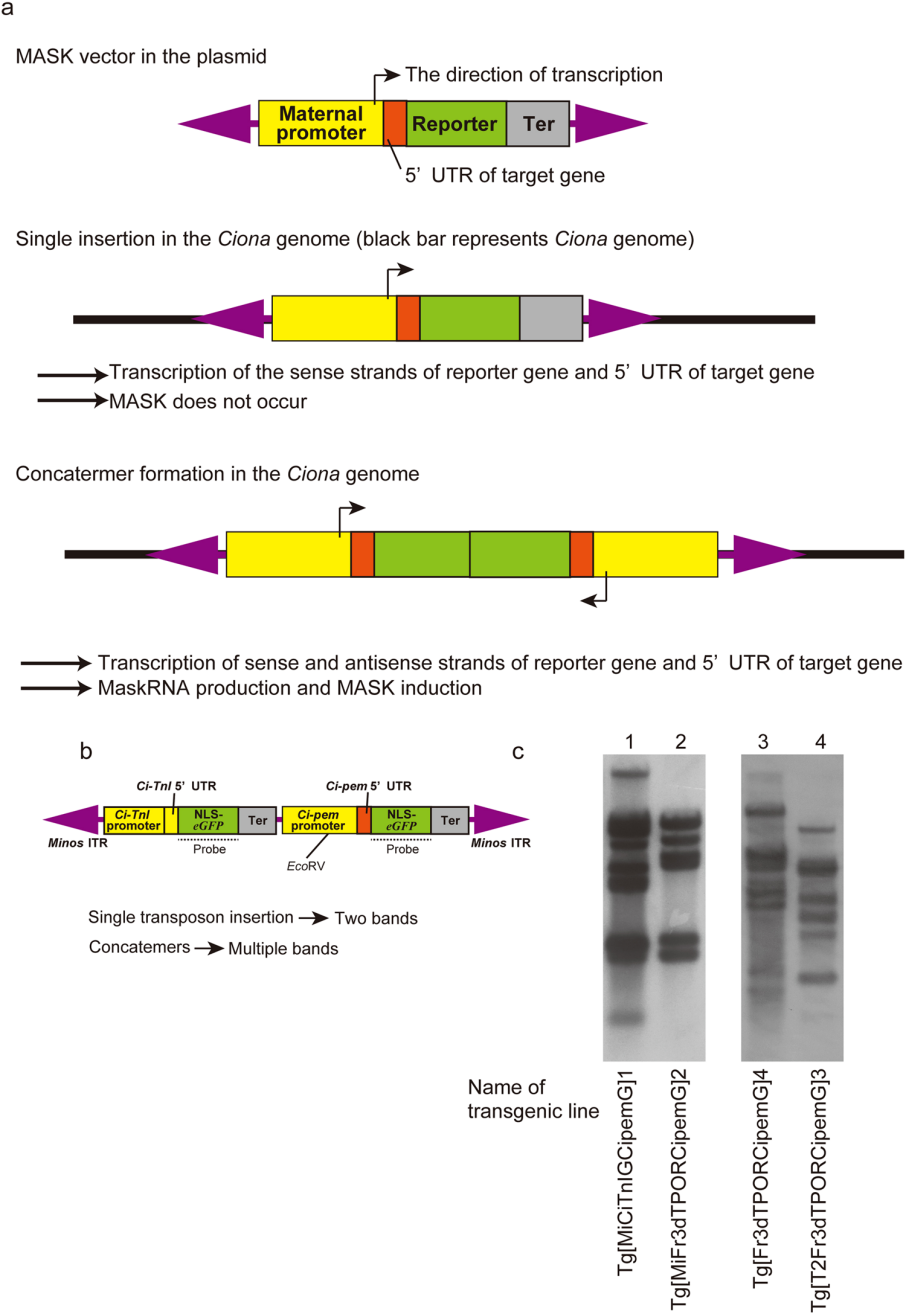
Mechanisms of the occurrence of MASK (maternal mRNA-specific knockdown). (**a**) Schematic illustration of the occurrence of MASK in the transgenic lines of a MASK vector. (**b**,**c**) Concatemers of transgenes in the MASK transgenic lines, as revealed by Southern blotting. Panel b shows the locations of the *Eco*RV restriction site in the pMiCiTnIGCipemG vector (see Fig. 1a) that was used to create Tg[MiCiTnIGCipemG]1, as an example of the experiment in panel c. Because the *eGFP* probe can hybridize with the left and right fragments of this vector (the probe in b), the single insertion of this vector will yield two bands by Southern blotting. The appearance of multiple bands as shown in (**c**) indicates the insertion of multiple vector elements at the single genomic position, suggesting the formation of concatemers. The examined transgenic lines are shown at the bottom of the panel. Lanes 1 and 2 were detected with *eGFP* probe, while lanes 3 and 4 were detected with *DsRed* probe. Tg[Fr3dTPORCipemG]4 and Tg[T2Fr3dTPORCipemG]3 respectively correspond to w/o transposon and *Sleeping beauty* in Fig. 2.

piRNAs are known to be produced from piRNA clusters^44^. There are three types of piRNA clusters, classified by the strands of DNAs subjected to transcription. Among them, the uni-strand cluster may not work in *Ciona* MaskRNA production, because not all transgenic lines of MASK vector exhibited the occurrence of MASK even though they have the same uni-directional transcription unit that transcribes sense strand mRNAs of the target gene and reporter gene (Fig. 6a). Rather, the dual-strand and/or bi-directional cluster may be more appropriate models to explain MASK.

In *Ciona*, the insertion of transposons often forms a concatemer of transgenes^25,50,51^. Such a concatemer may have a chance to mimic a dual-strand and/or bi-directional piRNA cluster by the rearranged transcriptional units (Fig. 6a). Indeed, all MASK-positive transgenic lines (n = 8) possessed a concatemerized transgene, as revealed by Southern blotting (Fig. 6b,c). Our MASK transgenic lines usually possess the transgene insertion at a single genomic site per line. If a single transposon vector were inserted into the insertion site, the transposon insertion would have yielded a limited number of bands by Southern blotting (Fig. 6b). However, the numbers of bands detected by Southern blotting were much greater than the expected numbers, indicating that multiple vector elements were inserted into the single genomic loci, and suggesting the formation of the concatemers. The formation of a piRNA cluster-like genetic element in the *Ciona* genome probably does not require a specific DNA sequence. This is in accordance with our present results showing that a specific DNA element is unnecessary for MASK vector. Moreover, the creation of an appropriate concatemer for the production of MaskRNAs may occur by chance, suggesting that MASK occurs in some transgenic lines that have MASK vectors as the transgene and that the vector copies are appropriately rearranged in their genome so as to mimic a piRNA cluster. This characteristic can explain why MASK could not be induced in all transgenic lines. The knockdown of genes in other loci by tandem arrays of transgenes was reported in the event named paramutation^52^. In the animal *Drosophila melanogaster*, piRNAs are produced from the tandem transgenes, which is essential for the paramutation^53^. Probably MASK in *Ciona* uses the mechanism similar to *Drosophila* paramutation.

The similarity between MaskRNAs and piRNAs suggests that the design of a MASK vector could be improved by mimicking the mechanism producing piRNAs. For example, the transcriptional unit of both sense and antisense strands of a part of the target maternal genes, like that shown in the bottom of Fig. 6, would greatly enhance the occurrence of MASK in all transgenic lines of the MASK vector. We could apply MASK to other tissues of *Ciona* where piRNAs could be produced. The testis is a strong candidate for such improvement because piRNAs are primarily produced in the testes in mice. In addition, MASK could be introduced into other organisms for facilitating functional analyses of maternal factors, since piRNA is observed in various metazoans^54^. We would also like to emphasize that the characterization of the mechanisms of a poorly-understood technique is fruitful for advancing the technique, as illustrated by our present findings regarding MASK.

## Methods

### Constructs

The open reading frames (ORFs) of *mKO2*^31^ and wild-type *GFP*^32^ were polymerase chain reaction (PCR)-amplified. The ORFs were subcloned into the *Bam*HI and *Eco*RI sites of pSPeGFP^25^ to create pSPmKO2 and pSPwtGFP. The 5′ upstream region including the 5′ UTR of *Ci-pem* was isolated by PCR. The PCR fragment was digested with *Bam*HI and subcloned into the *Bam*HI site of pSPKaede^55^, pSPNLS-DsRed^56^, pSPmKO2, and pSPwtGFP.

The fusion cassettes were subcloned into pMiFr3dTPORDestR^22^ using the Gateway^®^ technology (Invitrogen, Carlsbad, CA). Fr3dTPOR cassette was PCR-amplified, and the PCR product was subcloned into the *Bgl*II/*Eco*RV sites of pT2HB^57^ to create pT2Fr3dTPOR. The Gateway cassette was inserted into the *Eco*RV site of pT2Fr3dTPOR to create pT2RfB(R)Fr3dTPOR. *Ci-pem* > *NLS::eGFP* cassette was subcloned into pT2RfB(R)Fr3dTPOR using the Gateway technology. A Gateway cassette was subcloned into the blunted *Bgl*II site of pSPFr3dTPOR^56^ to create pSPFr3dTPORRfC1. *Ci-pem* > *NLSeGFP* cassette was subcloned into pSPFr3dTPORRfC1 using the Gateway technology. The official names of the vectors and transgenic lines according to the nomenclature rules for tunicates^58^ (Stolfi *et al*., 2015) were listed in Suppl. Table S5.

### Transgenic lines

The transgenic lines were created by transposon-mediated transgenesis or electroporation-mediated transgenesis as described^25,36,37^. Genomic DNA was isolated from the sperm of transgenic lines. The genomic DNA was digested with *Eco*RV, and Southern blotting was carried out according to a previous study^59^.

### Microinjection and *in situ* hybridization

*eGFP* mRNA was synthesized using the MEGAscript^®^ T3 kit (Ambion, Carlsbad, CA), the Poly(A) Tailing Kit (Ambion), and Cap structure analog (New England Biolabs, Ipswich, MA) as described^60^. We microinjected *eGFP* mRNA into unfertilized eggs derived from Tg[MiCiTnIGCipemG]2 or wild-type animals as described^61^. The microinjected unfertilized eggs were fertilized by sperm of counterpart animals so as to unify the genetic background. The concentration of mRNA in the injection medium was adjusted to 500 ngμl^−1^. After the embryos were fixed at the appropriate stage, whole-mount *in situ* hybridization (WISH) was performed as described^60,62^. The *eGFP* fluorescence was observed with a fluorescent microscope at the late tailbud stage.

### RNA-seq

We surgically isolated ovaries and mantle layers from well-grown *Ciona* adults of the transgenic lines. The ovaries were mashed with homogenizers in ISOGEN reagent (NipponGene, Tokyo). RNA was extracted according to the manufacturer’s instructions. After treatment with DNaseI, RNAs were subjected to phenol-chloroform and chloroform extraction, and then ethanol-precipitated. A 1-μg aliquot of each precipitated total RNA was then resuspended in 5 μl of nuclease-free water and used to construct a sequence library with the use of a TruSeq Small RNA Sample Prep Kit (Illumina, San Diego, CA) following the manufacturer’s instruction.

The amounts and sizes of the sequence libraries were measured on an Agilent 2100 Bioanalyzer with a DNA 1000 Chip (Agilent Technologies, Santa Clara, CA). Sequencing was performed on a HiSeq. 1500 (Illumina) or Miseq (Illumina) high throughput sequencer using a single-end 100-cycle run. Total reads were extracted with CASAVA v1.8.2 software (Illumina). The obtained sequences were uploaded in the Sequence Read Archive (SRA) (SRA ID: SRR6012511-SRR6012517; Suppl. Table S6).

Next, adaptor sequences and low-quality reads were removed from the extracted reads using the Trimmomatic 0.33 command line tool^63^. The remaining reads were then aligned using the Bowtie ver. 2.2.3 program^64^ allowing up to 20 multiple-hits to the reference sequences. The reference sequences were constructed by combining all of the plasmid sequences used for recombination and the *C. intestinalis* genome (KH, ver. 2008), which was downloaded from the Ghost Database^65^. The reads per megareads (RPM) value of each nucleotide in reference sequences was calculated by dividing the number of mapped reads (depth) for each nucleotide in the reference sequence calculated using samtools (ver. 1.3)^66^ by the total reads (M reads). The frequency of first residues and the length of the reads were calculated for the ORF region and the 5′ UTR region of *eGFP*, *Kaede*, *Ci-pem*, and *Ci-Nut*. The depth and frequency analyses were performed separately for sense RNAs and antisense RNAs.

## Electronic supplementary material

Supplementary Tables
