# Supplementary material for: piRNA-like small RNAs are responsible for the maternal-specific knockdown in the ascidian Ciona intestinalis Type A
Source: Sci Rep. 2018 Apr 12;8:5869. doi: 10.1038/s41598-018-24319-w (PMC5897368; doi:10.1038/s41598-018-24319-w)
Supplement: Supplementary file 1 — Supplementary Tables [file 41598_2018_24319_MOESM1_ESM.pdf]

Scientific Reports Supplementary information

piRNA-like small RNAs are responsible for the maternal-specific knockdown in the ascidian *Ciona intestinalis* Type A

Teruki Satoh[1]*, Takako Iitsuka[1]*, Akira Shiraishi[2]*, Akiko Hozumi[1], Honoo Satake[2], Yasunori Sasakura[1**]

[1]Shimoda Marine Research Center, University of Tsukuba, Shimoda, Shizuoka, Japan
[2]Bioorganic Research Institute, Suntory Foundation for Life Sciences, Kyoto, Japan

*These three authors contributed equally to this study.

Corresponding author: Prof. Yasunori Sasakura, Shimoda Marine Research Center, University of Tsukuba, 5-10-1 Shimoda, Shizuoka 415-0025, Japan.
sasakura@shimoda.tsukuba.ac.jp

Supplementary Table S1. Reporter gene requirement for MASK

Reporter gene	No. of *Ci-pem* knockdown lines	No. of examined transgenic lines
eGFP *	2	3
Kaede	4	6
DsRed	2	4
mKO2	2	2
Wild-type *GFP*	4	5

*This result was reported in [23].

SupplementaryTable S2. Small RNAs read from *eGFP* MASK lines.

Sample name	Total read number	*eGFP* ORF		*Ci-pem cis*		*Ci-pem* 5'UTR		*Ci-pem* ORF	
		Antisense (RPM)	Sense (RPM)	Antisense (RPM)	Sense (RPM)	Antisense (RPM)	Sense (RPM)	Antisense (RPM)	Sense (RPM)
Tg[MiCiTnIGCipemG]2 ovary	4,760,597	255.2	22.55	27.06	9.49	83.09	0.23	93.77	1.66
Tg[MiCiTnIGCipemG]1 ovary	1,445,439	91.51	3.97	4.97	5.96	6.96	0	5.96	1.98
Tg[MiCiTnIGCipemG]9 ovary	4,569,315	3.43	3	1.28	0.42	0	0	0.42	63.58
Tg[MiCiNutG]3 ovary	3,077,813	2117.11	457.07	0	0	0	0	2.41	2.41
Tg[MiCiNutG]4 ovary	15,296,994	0.68	0.98	1.27	0.68	0	0	0.88	1.27
Tg[MiCiTnIGCipemG]2 mantle	8,995,040	23.57	7.27	0.31	0.47	0	0	6.01	4.27

Table S2 (continued)

Sample name	*Ci-Nut cis*		*Ci-Nut* 5'UTR		*Ci-Nut* ORF	
	Antisense (RPM)	Sense (RPM)	Antisense (RPM)	Sense (RPM)	Antisense (RPM)	Sense (RPM)
Tg[MiCiTnIGCipemG]2 ovary	0	0	0	0	0	0.23
Tg[MiCiTnIGCipemG]1 ovary	0.99	0	0	0	0.99	0
Tg[MiCiTnIGCipemG]9 ovary	0	0	0	0	22.77	0
Tg[MiCiNutG]3 ovary	21.21	24.58	44.35	151.87	57.85	229.5
Tg[MiCiNutG]4 ovary	0	0	0	0	0.39	0.19
Tg[MiCiTnIGCipemG]2 mantle	0.15	0	0	0	0.15	0.63

Supplementary Table S3. Small RNAs read from the *Kaede* MASK line.

Sample name	Total read number	*Kaede*		*eGFP*		*Ci-pem cis*		*Ci-pem* 5'UTR		*Ci-pem* ORF		*DsRed*	
		Antisense (RPM)	Sense (RPM)	Antisense (RPM)	Sense (RPM)	Antisense (RPM)	Sense (RPM)	Antisense (RPM)	Sense (RPM)	Antisense (RPM)	Sense (RPM)	Antisense (RPM)	Sense (RPM)
Tg[MiFr3dTPORCipemK]4 ovary	3,350,166	82.54	86.94	4.4	4.95	6.6	30.26	3.85	0	1.65	0	284.49	1033.98

Supplementary Table S4. Length distribution of MaskRNAs

	% of MaskRNA							
	Tg[MiCiTnIGCipemG]2		Tg[MiCiTnIGCipemG]1		Tg[MiFr3dTPORCipemK]4		Tg[MiCiNutG]3	
Length in nucleotides	*eGFP* antisense	*Ci-pem* 5'UTR antisense	*eGFP* antisense	*Ci-pem* 5'UTR antisense	*Kaede* antisense	*Ci-pem* 5'UTR antisense	*eGFP* antisense	*Ci-Nut* 5'UTR antisense
23	1	0.5	0	0	2.7	0	1.6	7.7
24	1.6	0.2	0	0	4.7	16.6	7.1	10.3
25	3.2	0.5	0	0	5.4	0	12.3	18.1
26	5.6	0.8	2.2	14.2	11.5	33.3	9.2	31.1
27	9.5	2.3	10	14.2	8.8	16.6	11.1	12.9
28	30	43.6	42.2	28.5	45.5	16.6	14.2	7.7
29	23	32.5	21.1	28.5	5.4	16.6	17.6	3.8
30	20.1	14.6	17.7	14.2	8.8	0	18.9	3.8
31	3.8	4.1	6.6	0	4	0	6.2	2.5
32	1.7	0.2	0	0	2.7	0	1.2	1.2
No. of small RNAs	1031	341	90	7	147	6	4082	77

In order to see the distribution of the length of MaskRNAs in detail, we selectively extracted the data that correspond to MaskRNAs from Table 1 to create this table.

Supplementary Table S5. The full names of DNA constructs used in this study.

Figure number or section nar	Name in this manuscript	Full name according to the nomenclature rule
Fig. 1	*Kaede*	pMi-(Ciinte.REG.KH2012.C10.4438567-4440059|*Msi* :Ciinte.REG.KH2012.L3.178445-177583|*TPO* >NLS::*DsRed* ;Ciinte.REG.KH2012.C1.3314338-3315452|*pem* >*Kaede*)
	NLS-*DsRed*	pMi-(Ciinte.REG.KH2012.C10.4438567-4440059|*Msi* :Ciinte.REG.KH2012.L3.178445-177583|*TPO* >NLS::*DsRed* ;Ciinte.REG.KH2012.C1.3314338-3315452|*pem* >*NLS::DsRed*)
	NLS-*mKO2*	pMi-(Ciinte.REG.KH2012.C10.4438567-4440059|*Msi* :Ciinte.REG.KH2012.L3.178445-177583|*TPO* >NLS::*DsRed* ;Ciinte.REG.KH2012.C1.3314338-3315452|*pem* >*NLS::mKO2*)
	wild type *GFP*	pMi-(Ciinte.REG.KH2012.C10.4438567-4440059|*Msi* :Ciinte.REG.KH2012.L3.178445-177583|*TPO* >NLS::*DsRed* ;Ciinte.REG.KH2012.C1.3314338-3315452|*pem* >wt*GFP*)
Fig. 2	pT2Fr3dTPOR;CipemG	pT2-(Ciinte.REG.KH2012.C1.3314338-3315452|*pem* >*eGFP* ;Ciinte.REG.KH2012.C10.4438567-4440059|*Msi* :Ciinte.REG.KH2012.L3.178445-177583|*TPO* >NLS::*DsRed*)
	pSPFr3dTPOR;CipemG	pCiinte.REG.KH2012.C10.4438567-4440059|*Msi* :Ciinte.REG.KH2012.L3.178445-177583|*TPO* >NLS::*DsRed* ;Ciinte.REG.KH2012.C1.3314338-3315452|*pem* >e*GFP*
Methods	pT2Fr3dTPOR	pT2-(Ciinte.REG.KH2012.C10.4438567-4440059|*Msi* :Ciinte.REG.KH2012.L3.178445-177583|*TPO* >NLS::*DsRed*)
	pT2RfB(R)Fr3dTPOR	pT2-(RfB;Ciinte.REG.KH2012.C10.4438567-4440059|*Msi* :Ciinte.REG.KH2012.L3.178445-177583|*TPO* >NLS::*DsRed*)
	pSPFr3dTPORRfC1	pCiinte.REG.KH2012.C10.4438567-4440059|*Msi* :Ciinte.REG.KH2012.L3.178445-177583|*TPO* >NLS::*DsRed* ;RfC1

Supplementary Table S6. The correspondence between RNA seq data and SRA ID.

SRA ID	Transgenic line	organ
SRR6012511	Tg[MiCiNutG]3	ovary
SRR6012512	Tg[MiCiNutG]4	ovary
SRR6012513	Tg[MiFr3dTPORCipemK]4	ovary
SRR6012514	Tg[MiCiTnIGCipemG]1	ovary
SRR6012515	Tg[MiCiTnIGCipemG]2	ovary
SRR6012516	Tg[MiCiTnIGCipemG]2	mantle layer
SRR6012517	Tg[MiCiTnIGCipemG]9	ovary
